# Exploring Patients’ Experiences in a Blended Transdiagnostic Group Treatment: Qualitative Study

**DOI:** 10.2196/86016

**Published:** 2026-07-06

**Authors:** Noelia Jiménez-Orenga, Alba Fadrique-Jiménez, Azucena García-Palacios, Jorge Osma, Amanda Díaz-García, Juana Bretón-López

**Affiliations:** 1 Department of Basic, Clinical Psychology and Psychobiology, Universitat Jaume I, Castellón, Spain; 2 Department of Psychology and Sociology, Faculty of Social and Human Sciences, Universidad de Zaragoza, C/ Ciudad Escolar, 4, Teruel, 44003, Spain, 34 978 61 81 54, 34 978618154; 3 Health Research Institute of Aragon (IIS Aragón), Zaragoza, Spain; 4 CIBER de Physiopathology of Obesity and Nutrition (CIBEROBN), Instituto de Salud Carlos III, Madrid, Spain

**Keywords:** qualitative research, opinion, acceptability, completers, dropouts, transdiagnostic, CBT, group, blended, emotional disorders, anxiety, depression

## Abstract

**Background:**

Emotional disorders (EDs) are the most prevalent mental disorders worldwide. Health services face significant difficulties in attending to the high demand and applying evidence-based psychological treatments. Combining the transdiagnostic approach with the group and blended formats could help the accessibility of treatment for ED. It is important to assess the feasibility and acceptability of new interventions from a qualitative perspective.

**Objective:**

This study aimed to explore the experiences and opinions of patients with ED who have received transdiagnostic cognitive behavioral therapy treatment in a group and blended format.

**Methods:**

Two subsamples of participants were included: those who completed the intervention (n=18) and those who did not (n=4). The completers’ subsample participated in focus groups, which were transcribed verbatim and subsequently analyzed using a consensus qualitative research methodology. The noncompleters subsample completed a brief online questionnaire. Key themes identified by 2 independent researchers were described and used as complementary to focus group findings. Results were reported following the COREQ (Consolidated Criteria for Reporting Qualitative Research) guidelines.

**Results:**

After analyzing the focus group interviews using the consensus qualitative research method, 8 domains (or topic areas) were identified, namely experience with the online platform, configuration of the blended intervention, therapeutic content, experience with the group sessions via videoconference, role of the therapists, overall assessment of the blended treatment, elements that help maintain adherence, and suggestions for improvement. Overall, participants reported benefits from this type of intervention, expressed satisfaction with the treatment, and highlighted the perceived improvement and usefulness of what they learned. They also noted the good usability of the platform and the possibility of establishing a good therapeutic alliance in this format. Participants also pointed out unfavorable aspects and offered suggestions on possible areas for improvement. For noncompleters, various reasons for dropping out were identified: lack of treatment efficacy, intervention-related stress, difficulty in applying learned strategies, low involvement, lack of motivation, time constraints, or need for different types of help. Strategies to enhance adherence to treatment were also identified, such as changes in the format and configuration of the sessions, allowing more time to discuss individual problems or reducing the number of homework assignments.

**Conclusions:**

These qualitative results support the feasibility, acceptability, and clinical utility of a blended group transdiagnostic intervention for the treatment of ED. This novel format could be a scalable and well-valued option within mental health services, although it can still be further optimized based on the results of this study.

## Introduction

Depressive, anxiety, and other related disorders, commonly known as emotional disorders (EDs) [1], are the most prevalent mental disorders worldwide [2]. In Spain, the cases of ED reach almost 17% of the general population, being the most frequently diagnosed psychological disorders in health care services [3] and generating a high economic burden [4].

In this regard, the National Health System (NHS) faces significant challenges in meeting the high demand for mental health care, with a limited number of psychology professionals [5]. While international recommendations suggest a caseload of approximately 86 patients per year per professional, psychologists in the Spanish NHS attend approximately 330 patients annually [6]. This contributes to delayed care, long waiting lists, and extended time between sessions, thereby hindering the implementation of evidence-based treatments (EBTs).

To address these limitations, alternative therapeutic approaches with a more dimensional perspective have been proposed. The transdiagnostic approach stands out among them. This approach focuses on intervening in common factors involved in the origin and maintenance of various psychological disorders [7], such as the various EDs [8], which could facilitate the implementation of EBT and the management of comorbidities [9].

In parallel, digital mental health interventions, particularly internet-based therapy, have emerged to improve treatment accessibility. Online cognitive behavioral therapy (CBT) has been shown to be effective for treating anxiety and depression [10], even when using a transdiagnostic approach to address ED [11]. However, some patients require more therapist contact and customized treatment [12,13]. Blended treatments, which combine elements of face-to-face and online therapy, have emerged as a promising alternative, leveraging the strengths of both approaches while reducing some of their barriers [14]. Additionally, the blended format has been effective in reducing symptoms of depression and anxiety [15-17].

Another alternative that can help to improve psychological care, especially in the NHS, is to carry out interventions in group format. This type of intervention has been shown to be as effective as individual therapy in treating ED [18,19] and also has advantages for patients, such as peer support and reducing stigma [20,21]. Furthermore, it is a more efficient option than individual therapy as it allows treating many individuals at once and reducing the burden of services [21,22].

Consequently, there is growing interest in combining transdiagnostic, blended, and group approaches into a single treatment protocol [23]. This integration could help more people with ED access EBT and allow a greater number of individuals with similar issues to be treated simultaneously. However, beyond effectiveness, it is crucial to evaluate the feasibility and acceptability of these innovative interventions from both quantitative and qualitative perspectives [24]. Qualitative research is a valuable method for collecting and analyzing nonnumerical data, enabling an in-depth exploration of individual experiences and providing detailed information about concepts and opinions [25].

There are some qualitative studies on blended interventions from the patients’ [26,27] and the therapists’ points of view [28,29]. However, literature in this area remains scarce, and, to our knowledge, no studies have reported the opinion of patients who have received a transdiagnostic CBT intervention for ED applied in a blended and group format.

This qualitative study is part of a broader feasibility trial (trial registration: NCT04008576) [23] and its main objective was to explore in depth the experiences and opinions of patients who received a transdiagnostic CBT intervention in blended and group formats for ED, in order to understand their degree of acceptance, satisfaction, and possible areas for improvement from their perspective. Quantitative results on feasibility, acceptability, and preliminary efficacy are reported elsewhere [30].

## Methods

### Participants

Participants were selected through convenience sampling from a community sample of patients who had received a blended transdiagnostic group intervention for EDs at the Emotional Disorders Clinic of Universitat Jaume I [30]. Patients were required to be 18 years or older; no upper age limit was imposed. The initial sample included both individuals who had received psychological treatment prior to the feasibility trial and participants who were receiving psychological therapy for the first time.

Patients began the same intervention but differed in their level of treatment adherence; 23 out of 34 participants (68%) completed the full intervention, and 11 out of 34 (32%) discontinued treatment [30]. Therefore, this study included 2 subsamples based on treatment completion status. This was done to explore the experiences of those who finished the intervention and those who dropped out.

The first subsample consisted of 18 patients (14 women and 4 men) who completed the full intervention protocol, with a mean age of 33.94 (SD 11.03; range 20‐54) years. Sociodemographic and clinical characteristics of participants at the beginning of the treatment can be found in Table 1.

**Table 1.** Sociodemographic and clinical characteristics of subsample 1.

Code	Sex	Age	Marital status	Educational level	Main diagnosis	Comorbid diagnoses
P^a^1#FG^b^1	Female	23	In a relationship	HE^c^	MDD^d^	3 (PD^e^, A^f^, GAD^g^)
P2#FG1	Female	54	Married	HE	GAD	0
P3#FG1	Female	27	Single	HE	GAD	3 (MDD, PD, A)
P4#FG1	Male	35	In a relationship	HE	MDD	2 (GAD, SAD^h^)
P5#FG1	Female	38	Married	ME^i^	GAD	3 (MDD, SAD, A)
P6#FG1	Female	52	Married	HE	GAD	2 (OCD^j^, MDD)
P7#FG2	Female	23	In a relationship	HE	PDD^k^	1 (GAD)
P8#FG2	Female	23	In a relationship	HE	GAD	1 (MDD)
P9#FG2	Female	27	In a relationship	HE	NS anxiety disorder^l^	0
P10#FG2	Female	20	In a relationship	HE	GAD	3 (OCD, MDD, A)
P11#FG3	Male	40	Single	HE	A	2 (PD, GAD)
P12#FG3	Female	27	Single	HE	MDD	2 (SAD, GAD)
P13#FG3	Female	51	Married	HE	MDD	1 (GAD)
P14#FG4	Female	40	Married	EE^m^	A	2 (PD, SAD)
P15#FG4	Male	34	In a relationship	HE	GAD	1 (MDD)
P16#FG4	Female	35	In a relationship	HE	MDD	3 (GAD, SAD, A)
P17#FG4	Female	20	Single	HE	GAD	0
P18#FG4	Male	42	Single	HE	PD	3 (PDD, A, GAD)

a P: participant.

b FG: focus group.

c HE: higher education.

d MDD: major depressive disorder.

e PD: panic disorder.

f A: agoraphobia.

g GAD: generalized anxiety disorder.

h SAD: social anxiety disorder.

i ME: middle education.

j OCD: obsessive-compulsive disorder.

k PDD: persistent depressive disorder (dysthymia).

l NS anxiety disorder: nonspecified anxiety disorder.

m EE: elementary education.

The second subsample consisted of 4 patients (all women) who initiated but discontinued the intervention before completing it (dropout). Their mean age was 28.5 (SD 12.39; range 21‐47) years. Sociodemographic and clinical characteristics of these participants at the beginning of the intervention can be found in Table 2.

**Table 2.** Sociodemographic and clinical characteristics of subsample 2.

Code	Sex	Age	Marital status	Educational level	Main diagnosis	Comorbid diagnoses
D^a^1	Female	24	In a relationship	HE^b^	SAD^c^	1 (A^d^)
D2	Female	22	Single	HE	OCD^e^	1 (MDD^f^)
D3	Female	21	In a relationship	HE	SAD	1 (MDD)
D4	Female	47	In a relationship	HE	GAD^g^	3 (MDD, SAD, A)

a D: dropout.

b HE: higher education.

c SAD: social anxiety disorder.

d A: agoraphobia.

e OCD: obsessive-compulsive disorder.

f MDD: major depressive disorder.

g GAD: generalized anxiety disorder.

### Treatment

The treatment in which all participants previously took part was a transdiagnostic CBT intervention designed to address ED, composed of 16 modules and lasting 24 weeks. It was delivered in a group and blended format, so that patients attended a total of 8 group sessions by videoconference (2 hours in duration), and, in the period between sessions (3 weeks), they worked on the therapeutic content through online modules on a web platform. In this platform, the recommendation was to advance at a rate of approximately one module per week, allowing participants to consolidate therapeutic content between sessions. During the intervention, 2 messages of support per week were sent to participants (an email and an SMS text messaging). These messages served as reminders and motivational prompts to encourage continued participation. Further details regarding the intervention structure and content can be found in the study protocol [23] and in the paper on quantitative results [30].

### Procedures

The qualitative data collection procedures differed according to participants’ treatment completion status. On the one hand, those who completed the treatment (n=23) were invited to participate in focus groups (in-depth group opinion interviews). This was done with the goal that interview participants had sufficient knowledge about the intervention and could provide more complete opinions on all the aspects explored. Invitations were made during the final treatment sessions and by email. Finally, a total of 18 participants accepted and took part in the interviews (subsample 1), while the remaining 5 were unable to attend.

A total of 4 focus groups were conducted, one per treatment group, each lasting approximately one hour. Meetings were held via videoconference between 2 and 5 weeks after the end of treatment. All interviews were recorded and subsequently transcribed verbatim for later analysis. No field notes were taken.

Each focus group was conducted by 2 psychologists from the research team. In total, 5 psychologists (4 women and one man) conducted the interviews; 3 of them PhD students (NJ-O, Macarena Paredes-Mealla, and Rosa Lorente-Català) and 2 were PhDs in psychology with previous experience in qualitative research (AD-G and Alberto González-Robles). All interviewers were also experienced clinicians and researchers in transdiagnostic and internet-based interventions. These psychologists were the therapists for the therapy groups in the feasibility study, so to ensure an unbiased procedure, they only interviewed those participants who had not previously been their patients (eg, Macarena Paredes-Mealla and Alberto González-Robles moderated an interview with participants whose therapy group had been led by AD-G and NJ-O). During the focus groups, there were no observers; only the 2 interviewers and the participants were present. Participants were informed that the aim of the study was to explore their opinions and experiences in order to improve the future implementation of the intervention.

Focus groups continued until all available participants from the previous study had been interviewed, corresponding to one group interview per therapy group. The research team determined that data saturation had been reached. According to Krueger and Casey [31], a commonly accepted rule of thumb is to plan 3 or 4 focus groups for a specific audience and, once they have been conducted, determine whether data saturation has been reached, that is, the point at which ideas begin to be repeated and no new information emerges. Furthermore, in the consensus qualitative research (CQR) methodology, it is recommended to include at least 8‐15 participants to achieve data saturation [32,33].

On the other hand, participants who did not complete treatment (n=11) were invited via email to complete a brief open-ended online questionnaire aimed at exploring their reasons for dropping out. A total of 4 participants completed this questionnaire (subsample 2), while the remaining participants did not respond to the questionnaire nor to the email.

### Interview Protocol

Following recommendations [31,33], a semistructured interview with 12 open-ended questions was developed to explore participants’ perceptions and experiences in depth through focus group discussions. To that end, 2 doctoral students reviewed the literature of qualitative studies on psychological interventions, including those using blended treatment formats. Afterward, each doctoral student independently designed a list of relevant interview questions, which were then shared and discussed collaboratively until consensus was reached. Finally, 2 senior researchers (AD-G; JB-L) and full professors reviewed the proposal of questions, and the final version was defined. An effort was made to reach a balance between obtaining the greatest amount of information possible and asking the fewest number of questions. The interview script can be found in Multimedia Appendix 1. Two questions that constituted the dropout questionnaire can be seen below in Table 3.

**Table 3.** Responses to the dropout questionnaire.

Participant	Responses	
	Question 1: Please write a comment indicating the reasons why you have discontinued the treatment.	Question 2: Please write a comment indicating any strategy that, in your opinion, could help to improve the treatment or enhance adherence to it.
D^a^1	“It hasn’t helped me and it overwhelmed me more than it helped me.”	“That it be individual or that it doesn’t have so many mandatory tasks.”
D2	“I wasn’t fully committed, and since I thought this study was helpful in a more general way, I thought in-person therapy would be more effective for me.”	“I think it would help if the sessions were in person, although that would mean the study could only be conducted with people from Castellón. Also, sometimes I didn’t know how or at exactly what moments to apply the treatment, and I didn’t know where to start.”
D3	“I’ve gotten worse and I’m not able to do things on my own. My excessive lack of motivation and energy prevented me from studying and applying the tools I was given. That’s why I decided I needed more specialized help, because I’m not able to follow through on my own.”	“It could be improved by dedicating part of the group session to sharing problems that are currently worrying each person and talking about them a bit with everyone.”
D4	“Lack of time to follow treatment guidelines.”	“Well, I’m not sure, but maybe, personally, weekly in-person sessions would help me get more organized. With so many things going on in my daily life, I still had to access the platform, and by the time I realized it, 3 weeks had already passed.”

a D: dropout.

### Data Analysis

The CQR methodology [32,33], which is a structured and rigorous method for analyzing qualitative information, was used to analyze the focus group data. This methodology combines elements from constructionism and from postpositivism, and seeks to understand people’s subjective reality while simultaneously reporting that reality as objectively as possible [33].

Two doctoral students trained in qualitative analysis acted as judges (NJ-O and AF-J), and 2 full professors with prior experience in qualitative research acted as auditors (AD-G and JB-L). All researchers involved in the analysis were women. One of the judges also served as an interviewer in the study.

First, the 2 judges independently identified the domains and then pooled them until a consensus was reached. The 2 auditors reviewed the proposed domains and again a consensus was reached between all of them. Subsequently, the judges independently identified and placed the core ideas from each interview into the corresponding domains and then shared them until a consensus was reached. Core ideas were also shared interview by interview with the auditors, and the necessary modifications were made until consensus was reached again. Finally, cross-analysis was carried out and the categories were established, following the same consensus procedure explained above. The information has been identified from the data (data-driven coding), although the domains partially correspond to the interview questions.

Following the suggestions of Hill et al [33], the frequency of each category was quantified to indicate its representativeness. Categories were labeled as “General” if they applied to all or all but one case (17‐18 cases), as “Typical” if they applied to more than half of the cases (10‐16 cases), as “Variant” if they applied to half or fewer of the cases (3‐9 cases), and as “Rare” if they applied to only 1 or 2 cases.

The information available regarding the reasons for the dropping out of the participants who discontinued the intervention was very limited, so it was not considered appropriate to conduct a formal qualitative analysis based on an established methodology. Instead, this information was treated descriptively as complementary to the insights gathered in the focus groups. Nonetheless, the key themes identified independently by the 2 judges are briefly reported.

The COREQ (Consolidated Criteria for Reporting Qualitative Research; Checklist 1) [34] guideline was used to report the results.

### Ethical Considerations

The study received approval from the Ethics Committee of Universitat Jaume I (CD/40/2019) and all participants provided informed consent prior to participation. Participant data were handled in accordance with applicable data protection regulations. Privacy and confidentiality were ensured by anonymizing all data and storing them securely on password-protected institutional servers accessible only to the research team. No personally identifiable information is reported in the manuscript. Participants did not receive financial compensation for their participation in the study.

## Results

### Focus Group Results

A total of 8 domains and 47 categories were identified after analysis using the CQR method, 10 of which were categorized as “Typical,” 31 as “Variant,” and 6 as “Rare.” The results obtained from the focus group interviews are shown in Table 4, which includes several illustrative core ideas according to the frequency of the category.

**Table 4.** Results from focus group interviews.

Domains and categories (frequency)	Illustrative core ideas
Experience with the online platform	
Overall positive perception; typical (10)	P^a^4#FG^b^1 says he has had a good experience with the platform P13#FG3 indicates that she likes the platform P14#FG4 notes that the experience with the platform has been very positive for her and that it has been very good
Usability; variant (9)	P9#FG2 highlights that the platform is very easy to use P12#FG3 indicates that she found the platform to be quite simple, intuitive, and easy to use P15#FG4 considers that it has been a simple and easy-to-use platform
Availability of resources and content; variant (7)	P5#FG1 appreciates having a tool with all the contents, multimedia, tasks, and monitoring logs P10#FG2 says that one of the advantages is being able to review all the resources once the treatment is over
Positive assessment of the tools offered; variant (6)	P12#FG3 appreciates having PDF summaries at the end of the modules because it is like a “photograph” that helps her to review P16#FG4 adds that being able to download the PDFs and have them at hand to implement the strategies has been a key point and that she was very pleased to be able to see the progress graphs
Utility; rare (2)	P9#FG2 reports that the platform has been very useful to complement what was seen in the sessions
Difficulties; variant (7)	P5#FG1 agrees with the disconnection of the platform during use and indicates annoyance at not being able to access the specific part of the module where she had reached P8#FG2 mentioned that the “calendar” tool did not indicate the days she had accessed the platform
Configuration of the blended intervention	
Positive aspects (pace of progress, frequency and duration of sessions); typical (14)	P1#FG1 says she feels it is a good pace to advance at one module per week and feels it works P10#FG2 notes that if the duration of the sessions had been longer, they would have been tedious, and if they had been shorter, there would not have been enough time to deal with all the topics P13#FG3 is satisfied with the timing, she thought it was good that the contents were explained in the sessions and then they had a 3-week period to work on them, she thinks it was adequate P16#FG4 commented that she has found the length of the sessions and the spacing between them to be very good
Unfavorable aspects (demand, frequency and duration of sessions); typical (10)	P3#FG1 said that the duration of the sessions was a bit long and tiring P4#FG1 expresses that he felt that the waiting time until the next session was too long and that he would have liked them to be more frequent P10#FG2 indicates that some modules were longer and required a little more time
Ambivalent opinion on the duration of the intervention; rare (2)	P18#FG4 points out that it is a long therapy and it is possible to fall behind and lose the motivation to continue
Individual variables that modulate the adequacy of the configuration; variant (7)	P8#FG2 explains that during the first modules she was a bit down in the dumps and found it hard to feel motivated to do them, although this did not pose a problem for her P13#FG3 indicates that her problem has been her lack of time to keep up with the platform and complete the tasks due to her busy life
Therapeutic contents	
Suitability and richness of the contents; typical (14)	P5#FG1 notes that there is a lot of information and many strategies that are useful P6#FG1 says that he really liked the content and finds it very useful, and agrees that everyone should have access to this syllabus P8#FG2 considers that the contents are very well explained and that it is a very complete program P9#FG2 considers that the richness lies in the fact that different strategies are provided and you can choose the one that best suits you
Notable usefulness of the different therapeutic components; typical (11)	P8#FG2 points out that the strategy that has helped her the most is cognitive flexibility P12#FG3 says that some modules have helped her more than others, especially those that are “action-oriented,” such as mindfulness and those related to positive affect P18#FG4 highlights the usefulness of mindfulness in his case and explains that he liked the exercises very much and noticed a difference when practicing them regularly
Difficulty in choosing strategies; rare (2)	P4#FG1 agrees that he lacked clarity on when to use each strategy
Influence of the level of prior knowledge about the contents; variant (4)	P4#FG1 mentions feeling overwhelmed by the amount of information due to his lack of prior knowledge
Experience with group sessions via videoconference	
Group bonding; typical (11)	P6#FG1 mentions looking forward to the session time to talk to everyone P15#FG4 considers that the exchange has helped them a lot to create synergies and that the group has also served as social support P17#FG4 points out feeling comforted because her peers said things that she had been through or she could say things that they could also identify with, and highlights that she would recommend group therapy because you feel more understood and a bond is generated
Positive aspects of the group format; typical (11)	P6#FG1 positively values being able to share with other people and that it helps to not feel lonely and strange P7#FG2 mentions being able to share your problems with others as an advantage P10#FG2 adds that an advantage of group therapy is learning a lot from the other participants, seeing how others deal with and manage their problems
Initial reluctance toward the group format; variant (7)	P7#FG2 acknowledges that when group therapy was proposed to her, she did not like the idea because she had a previous experience in which she had not felt well P15#FG4 notes that the first day he was very nervous and self-conscious, because he didn’t know what to say and if he could open up so easily
Limitations of the group format; variant (3)	P3#FG1 believes that the group format may limit the ability to share certain topics that could be shared privately
Relevant variables for the proper functioning of the group; variant (4)	P2#FG1 believes that they were a very heterogeneous group in terms of problems and life stages, and that it would have been better to find people with greater homogeneity
Positive aspects of videoconferencing; variant (8)	P3#FG1 appreciates the fact that the sessions were online since she would not have been able to attend in person due to the geographical distance P9#FG2 is grateful that the sessions were held via videoconference because wherever you are, you can connect to the session
Limitations of videoconferencing; variant (7)	P1#FG1 believes there may be connection problems and that this will determine the course of the session P7#FG2 agrees on the difficulty in expressing what she really felt and thought if there was someone at home
Preference for individual format; variant (5)	P2#FG1 expresses preference for individual treatment P13#FG3 shares that she prefers individual therapy that, since everyone has their own story, she likes more individual therapy because of the way she wants to work on her problem
Preference for in-person format; variant (8)	P14#FG4 mentions that it was difficult for her to speak via videoconference and that she would have preferred in-person format P17#FG4 points out that she prefers face-to-face versus online sessions, but believes that this aspect has been managed quite well in the sessions and was not bad
Satisfaction with the sessions; variant (4)	P9#FG2 mentions that she would always prefer the group format via videoconference and would recommend it that way because it worked very well for her, and also explains that online group therapy allows her to feel less exposed and invaded as well as to have freedom to intervene and to be able to be in a familiar environment during the sessions
Positive assessment of the dynamics followed in the sessions; rare (2)	P15#FG4 notes that reviewing previous modules at the beginning of the sessions helped refresh and remember what had been seen previously and made it easier to solve doubts and stay up to date
Role of therapists	
Positive evaluation of the therapists (abilities, aptitudes, and availability); typical (12)	P5#FG1 highlights that the therapists are wonderful, they have listened to them, explained to them, and emphasized when they had doubts P7#FG2 agrees that the therapists are very understanding, and highlights that they always gave each one time to talk and encouraged them to share P12#FG3 highlights that the therapists were always available when you asked them and responded fairly quickly and in detail P18#FG4 describes the therapists as excellent people, emphasizes their warmth and their excellent organization and preparation, and is very grateful for the availability of them to help and answer questions
Importance of therapist support; variant (9)	P5#FG1 considers the role of therapists to be fundamental; she believes that they provide the professional vision, help you to talk about certain topics, and make recommendations P9#FG2 considers that without the therapists they could not have done it and highlights that their support was vital throughout the process P11#FG3 believes that the support from the therapists has been good and that it always goes well; he explains that the therapists have been supportive for some things that they did not know or did not know how to interpret well
Establishment of a good therapeutic alliance; variant (9)	P6#FG1 reported having felt a bond with the therapists P10#FG2 agrees that the therapeutic alliance can be formed in the same way (as in face-to-face individual therapy) and considers that the commitment of the therapists to the patients and also of the patients to them and to the therapy has been noted P17#FG4 mentions that they have had a very good alliance with the therapists
Greater difficulty in creating the bond; variant (3)	P4#FG1 considers that in group it is more difficult to create a bond with the therapists, and that a different type of bond is created compared to individual therapy
Overall assessment of the blended treatment	
Perceived improvements and usefulness of learning; typical (15)	P5#FG1 indicates that she has learned many things and has found new methods that work for her P8#FG2 considers that she has changed a lot since the intervention and that it has helped her a lot, and adds that, although her life circumstances have not changed, she feels that her coping has improved tremendously P9#FG2 indicates that at the beginning she did not expect to get so many results and explains that thanks to the intervention her anxiety has decreased and she has been able to learn to manage crises, understand them, and know what to do when they occur P11#FG3 agrees that treatment has been useful to him, especially finding ways to deal with the problems that arise
Satisfaction with the treatment; variant (7)	P1#FG1 considers that the intervention has satisfactorily fulfilled everything expected in 70‐80% P10#FG2 is very grateful and happy for the intervention received, she values her evolution very positively and emphasizes that most of her initial objectives have been met
Recommended treatment; variant (5)	P7#FG2 notes that she would recommend this treatment to other people with emotional disorders P12#FG3 mentions that she would recommend the intervention and would invite anyone with an emotional disorder to do it because she believes it is really useful
Advantages; typical (11)	P11#FG3 considers that the combination of online content and sessions with the therapists has been very helpful and worth doing this way P8#FG2 highlights the temporal flexibility that this format allows and adds the spatial flexibility as an advantage, in the sense of being able to do the intervention from any location, which allows it to continue despite inconveniences or unforeseen circumstances such as COVID P18#FG4 adds that the main advantage he enjoyed was accessing the treatment from another country, explaining that otherwise he could not have done it and that it does not exist in his country, so for him it is a great advantage
Disadvantages; variant (9)	P5#FG1 indicates that the treatment takes too long because you have to know all the strategies to know which one you can use in your case P6#FG1 indicates that the treatment was more general and does not focus on what she believes her problem is P10#FG2 believes that this intervention format, in comparison to traditional therapy, does not allow to delve into the why of the problems or to understand where they come from at the individual level
Elements that help to maintain adherence	
Therapist support; variant (8)	P13#FG3 adds that the fact that 2 psychologists explain the topics helps to continue, she considers it as the main thing. A total of 2 psychologists explain the topics help to continue; she considers it as the main thing P18#FG4 points out that what helped him not to drop out despite being behind was that the therapists continued to invite him to attend the sessions, which gave him confidence
Therapeutic resources and materials; variant (6)	P13#FG3 mentions that the videos have helped her a lot to get motivated and continue with the treatment because she saw herself reflected in it P17#FG4 indicates that in moments of overwhelm or low mood, having the decisional balance table visible helped her a lot to move forward
Having graphs that represent progress; variant (5)	P1#FG1 highlights the progress graphs on the platform as motivating elements P16#FG4 mentions that seeing her week-by-week progress on the platform’s graphs helped motivate her to continue with the treatment
Having scheduled sessions; variant (5)	P9#FG2 highlights that the schedule of having to meet every 3 weeks for the sessions is what helped her stay consistent with the tasks, keep going, and complete the treatment P12#FG3 highlights the fact that there are established sessions as a motivating element that helps to keep going
Commitment to the group; variant (3)	P6#FG1 indicates that her groupmates are one of the elements that helped her the most to continue with treatment
Perceived impact of the intervention; variant (3)	P18#FG4 mentions that what motivated him the most was seeing that the tools were working, that watching the videos and applying the strategies really had an effect on his mood
Readiness to change; rare (2)	P5#FG1 highlights the desire to change as a person and to find new ways of doing things as a motivating element
Having reminder messages; rare (1)	P12#FG3 adds that the constant reminder messages, although subtle, have been very important for her in keeping going
Proposals for improvement	
Modifications to the platform; variant (6)	P1#FG1 suggests that the platform could be more interactive and incorporate activities that allow decision-making to be put into practice P16#FG4 notes that she has missed a forum or a similar tool to be able to use between sessions and try to make the group closer
Adjustments in the configuration (pace of progress, duration and periodicity of sessions); variant (5)	P3#FG1 suggests that sessions of one or one and a half hours and weekly frequency would be more bearable P11#FG3 notes that it would have been better if the time between sessions had been adapted according to the content to be worked on in the corresponding modules
Suggested changes regarding the sessions; variant (3)	P1#FG1 proposes doing some of the group sessions in person
Greater individualization of the intervention; variant (4)	P8#FG2 suggests having, from time to time, an individual space with the therapist for about 10 minutes to delve deeper into individual difficulties
Suggestions for improving adherence; variant (7)	P3#FG1 suggests that individualized weekly follow-up, including by email, could help improve treatment adherence P18#FG4 proposes leaving less space between sessions and doing them every 2 weeks to improve adherence for people who need more follow-up or who are a little disorganized

a P: participant.

b FG: focus group.

#### Experience With the Online Platform

This domain includes all experiences and opinions related to the online platform. It does not include what is clearly identified as proposals for improvement of the platform (included in the domain “proposals for improvement”). The categories found are described and exemplified below.

##### Overall Positive Perception (Typical)

Participants expressed an overall positive experience with the use of the platform. We can see an example in the following excerpt:

*Let’s see, for me the experience (with the platform) has also been very positive. And as my colleagues say, you had it there and you could go in and do it little by little. And the truth is that yes, it was very good. I liked it*.

[Participant (P) 14#focus group (FG) 4]

##### Usability (Variant)

Participants highlighted the simplicity of the platform and its ease of use. An example of this is the following extract:

*As for the use of the platform, I found it to be a fairly simple and intuitive platform, uh well, easy to use, also… all the different elements were quite clear, simple*.

[P14#FG4]

##### Availability of Resources and Content (Variant)

Some participants highlighted having the contents accessible through the platform as positive.

*I imagine in individualized therapy it won’t work the same way, that it will be totally different, they won’t give you… an application where you can read all the information, watch videos, do exercises, monitoring logs… so in that sense I think that… I liked it, it works well*.

[P5#FG1]

##### Positive Assessment of the Tools Offered (Variant)

Some participants appreciated the different tools found on the platform (eg, videos, downloadable PDFs, review tool for revisiting completed modules, and progress graphs).

*Eh, I’d add that for me a point that I really liked about the application is, eh, the section called “Weekly evolution” or something like that, the fact of physically seeing, that is, graphically seeing the evolution you’ve been having week by week. […] And besides, as P15#FG4 had said at the beginning, being able to download the PDFs later to have them at hand to be able to put them into practice in case you forget something or whatever, I also think that has been a key point.*

[P16#FG4]

##### Utility (Rare)

Some participants referred to the usefulness of the platform from a clinical point of view. The following fragment can serve as an example:

*And… I think it (the platform) has helped us a lot to complement everything that was discussed in each session.*

[P9#FG2]

##### Difficulties (Variant)

Participants mentioned perceived technical difficulties or aspects that they would have liked to be another way. We can see an example below:

*Eh, it’s true that when you went a while without using it, it disconnected immediately, you had to re-enter the program and what really bothered me the most was that maybe I didn’t have time to see the entire module and I only viewed maybe a couple of pages, and when I connected another day and re-entered, it wasn’t saved where I had left off, you had to start the module again and look for where you had left off. For me, this is the inconvenience that has caused me the most discomfort, but otherwise, in general, very well.*

[P5#FG1]

### Configuration of the Blended Intervention

This domain includes all aspects related to the structure of the blended intervention and how its different components were combined or organized.

#### Positive Aspects (Typical)

Most participants identified positive aspects of the configuration regarding the pace of progress on the platform, the frequency of the sessions, and the duration of the sessions.

*As for me, eh, I think the same as you. I thought they were good, the sessions were also quite good. If they had lasted longer, they would have been a bit tedious, and if they had been shorter, there would have been a lack of topics to address.*

[P10#FG2]

#### Unfavorable Aspects (Typical)

Participants also identified unfavorable aspects of the configuration in relation to the demand of the pace of progress as well as the periodicity and duration of sessions.

*It seemed like too much time to me. I would have liked a little more, closer together, but well, I understand that that’s what we had, but for me the group sessions were what helped me the most, and I would have liked them to be more frequent, and the duration of 2 hours seemed fine to me.*

[P4#FG1]

#### Ambivalent Opinion on the Duration of the Intervention (Rare)

Some participants expressed their opinion about the duration of the treatment. While one person pointed to the duration as a positive aspect, another person pointed to it as a more unfavorable aspect:

*The fact that it has lasted so long, for so many months, I think it has helped us a lot, yes, it has not been short, you see your progress month after month.*

[P9#FG2]

*That can happen, you can fall behind because it’s a long course, a long therapy, so you lose a little bit, you can also lose the motivation to keep going if you’re too far behind.*

[P18#FG4]

#### Individual Variables that Modulate the Adequacy of the Configuration (Variable)

Some participants also mentioned personal variables that may influence the appropriateness of the configuration in each case. The following excerpt is an example of this:

*My problem is that lately I’ve had a very busy life, but just as I haven’t been able to do my homework, I had to go to the physiotherapist and I haven’t gone, so I’m not justifying, but well, for failing something, it has been my time*.

[P12#FG3]

### Therapeutic Contents

This section includes all the experiences and opinions regarding the treatment modules or therapeutic strategies, which were taught both in the group sessions and on the online platform. The categories found are described and exemplified below.

#### Suitability and Richness of the Contents (Typical)

Most participants emphasized the adequacy of the contents as well as the richness of the program. We can see an example in the following extract:

*Personally, I thought the program was super complete, I really liked the fact that different tools and methodologies are provided because that way everyone chooses, tests, and at the end looks or explores what works best for him/her, and it is very well explained.*

[P8#FG2]

#### Notable Usefulness of the Different Therapeutic Components (Typical)

Participants highlighted those activities, strategies, or therapeutic components that they had found most useful in their case.

*Yes, in my case the same, mindfulness. I think, like everyone else, I really liked the exercises, I did the exercises as they came and yes, I noticed a difference. After about 3 or 4 days, I noticed a difference, and I think that if you do it constantly, it really helps you to relax. What other one? I also liked the cognitive flexibility one a lot, to see a fact from different perspectives, because one normally goes with the belief… that one already has programmed, right? The beliefs, distressing or anxious beliefs usually. So, trying to see it from different perspectives, that’s very useful. Very useful. And the identification of maladaptive behaviors as well, that normally one acts automatically, right? Again, according to the anxious perspective that you have, and you don’t realize that those behaviors are generating again the anxiety and the anxiousness, reinforcing the anxiety, the anxiousness. So those 3. I would highlight 3 very useful tools.*

[P18#FG4]

#### Difficulty in Choosing Strategies (Rare)

Some participants mentioned difficulty in knowing when or in which situations to use each of the strategies. An example of this is the following intervention:

*I haven’t been able to grasp, understand everything and so on, and especially what P5#FG1 was saying, that I don’t know when to use each thing, and that’s what really… I fell short on, I missed that part I think, of knowing when to use each thing and so on*.

[P4#FG1]

#### Influence of the Level of Prior Knowledge About the Contents (Variant)

Some participants indicated how their prior knowledge of the treatment contents influenced their evaluation. While some emphasized the importance of having some background, for others, having more prior knowledge made the contents seem somewhat repetitive:

*What happened to me was that… it overwhelmed me a little. I think the information was very good and all, but the thing is that since I didn’t have much (prior) knowledge, I suppose it overwhelmed me a little*

[P4#FG1]

*Eh, for me it was a bit the opposite, I informed myself a lot beforehand, so there were many things that felt very obvious or very repetitive to me. Well, I guess it depends on the person and their knowledge about the topic. And overall, well, yes, I obviously learned a lot of new things, and I also enjoyed that part… but yeah, in general, some of the information felt a bit boring because… I already knew it beforehand.*

[P3#FG1]

### Experience With Group Sessions via Videoconference

All experiences and opinions related to group sessions by videoconference are included here. Aspects related to the frequency and/or duration of the sessions, already included in the configuration domain, are not included in this section.

#### Group Bonding (Typical)

Participants reported a connection or bond with the group, which they identified as a source of support or understanding and acknowledged looking forward to the sessions to talk to everyone and share. The following excerpt is an example of this category:

*It comforted me because sometimes my groupmates would say things that I had also gone through, or I could say things that they could identify with. So, I think it has been quite good and it has changed my perspective. And what’s more, I would recommend it because you feel more understood and maybe there’s a bond of mmm I don’t know, there’s a bond there.*

[P17#FG4]

#### Positive Aspects of the Group Format (Typical)

Participants highlighted advantages or positive aspects of the group format, as exemplified in the following fragment:

*I think you also learn a lot. I mean, I think there are things I wouldn’t have learned if the others had not been there, because… everyone has their own experiences and their own way of dealing with things, and seeing how other people deal with the problem, even if it’s not included in the modules, or whatever, also helps a lot, because you can try it that way or not, and the truth is that in an individual therapy you don’t get that, that is, having other examples from real life, seeing a person who is doing it and living it, it helps a lot.*

[P10#FG2]

#### Initial Reluctance Toward the Group Format (Variant)

Some participants admitted to being uncertain about the group format and expressed initial feelings of fear, strangeness, or nervousness about having to speak in front of other people.

*At the beginning, before starting the group therapy, I was looking for individual therapy, but they recommended me ‘there is this group that is going to be formed, I think you would be interested in joining, try it, you don’t lose anything’, obviously. And it’s true that the first day I was very nervous, I was very self-conscious, like ‘I don’t know what to say, I don’t know if I can open up so easily’. But in the end, you mention your problem, you see that another person comments on his/her problem, there are points in common, what I can say can help a person or what another person says can help me, and I believe that throughout the sessions it has also been seen that we were talking more and more, it was less difficult for us to start talking.*

[P15#FG4]

#### Limitations of the Group Format (Variant)

A few participants also highlighted the possible limitations of the group format, as we can see in the following excerpt:

*I haven’t had experience of other types of therapy either, but I think that there are certain things that you might not be able to share in a group and that maybe in private you would be able to. I don’t know. That’s what I believe*.

[P3#FG1]

#### Relevant Variables for the Proper Functioning of the Group (Variant)

Some participants mentioned some important factors that may influence the group therapy experience, such as how the particular group gets along, how homogeneous the group is, or how participative people are:

*Well, I think that ehh I agree with everything my groupmates are saying, but I think that we were a very heterogeneous group. Ehh each one had a different problem, was in a different stage of life,… so… […] Maybe it would have been better to find groups of people among whom… people among whom there would have been a greater homogeneity, maybe.*

[P2#FG1]

#### Positive Aspects of Videoconferencing (Variant)

Participants highlighted advantages or positive aspects of the videoconference, as exemplified in the following extract:

*I really appreciated the fact that they are by videoconference, because each one has his/her own life rhythms and schedules, and no matter where you are in the world, you can connect 2 hours for the session.*

[P9#FG2]

#### Limitations of Videoconferencing (Variant)

Participants also highlighted possible limitations of conducting the sessions via videoconference, as we can see in the following intervention:

*The same as P8#FG2, that many times I have wanted to express what I felt, but like for example, I didn’t know if my roommates were there, I couldn’t say everything I really thought for fear of being heard.*

[P7#FG2]

#### Preference for Individual Format (Variant)

Some participants expressed a preference for individual therapy, as can be seen in this case:

*Well, I’m going to give my opinion first because I’ve gone through individual and group, and I think it is, I can be more objective. I prefer individual. I also like to have met mm the groupmates, but it’s true that as everyone has their own story… I like it more individual. I do… but that doesn’t mean group was bad for me, nooo, but I liked individual more, maybe because of the way I want to work on my problem.*

[P13#FG3]

#### Preference for In-Person Format (Variant)

Some participants expressed a preference for the in situ format, as exemplified in the following excerpt:

*Eh. I, like P15#FG4, was also looking for something individual, and they offered me this and I said I’m going to try it. And the truth is that it’s good, but I would prefer face-to-face. […] In videoconference I have felt that it’s more difficult for me to talk. The truth is that I have found it difficult to speak. Maybe it’s personal. I don’t know, it’s because I’m like that, but the truth is that face-to-face would have been better.*

[P14#FG4]

#### Satisfaction With the Sessions (Variant)

Some participants highlighted their satisfaction with the sessions received, as we can see in this fragment:

*I would add the fact that I would always prefer to do it online and in group rather than face-to-face and individual because in the individual sessions I’ve done I feel very exposed, very invaded, like now is the moment and you have to talk, say everything, you have this schedule, everybody, you know? Doing it in a group online, things come up, right now I don’t feel like talking, I listen to the other, you’re in your space, in your home, you’re not in a psychologist’s office, it’s different, I… I think I would always recommend doing it this way. It worked really well for me.*

[P9#FG2]

#### Positive Assessment of the Dynamics Followed in the Sessions (Rare)

Some participants positively valued the dynamics followed in the development of the group sessions via videoconference, for example:

*And also one thing we did in the sessions, which I imagine we’ll discuss later, was that at the beginning of the session, for example, the beginning of session 2, we reviewed a little bit what we had seen in the previous modules, so that also helped you refresh and remember what you had seen, in case there was something you had missed or you had doubts about, that also helped you to keep up to date. […] It’s also true that the dynamics within the sessions made everything quite, quite free-flowing and the feeling of tiredness wasn’t so common.*

[P15#FG4]

### Role of Therapists

This domain encompasses all the experiences, assessments, and opinions regarding the role of the therapists in the intervention, including therapeutic support received, therapeutic alliance, etc. The identified categories are outlined and illustrated below.

#### Positive Evaluation of the Therapists (Typical)

Participants positively rated the therapists in terms of their abilities, their aptitudes, and the availability shown throughout the intervention.

*Yes, apart from that, I totally agree with my groupmates, for example, they always gave each one of us our own time to talk, if for example at a certain moment we didn’t have time to talk, they always told us, ‘oh, how about you?’, and I don’t know, they are very understanding, really, we are very grateful that they have been there.*

[P7#FG2]

#### Importance of Therapist Support (Variant)

Participants emphasized the importance of having therapeutic support, that is, the presence of the therapists. We can see an example in the following excerpt:

*I think it’s fundamental, I mean, the role of therapists in this case. They are the ones who open you up to talk about certain topics, who help you, who… advise you, who give you their point of view, who is the… the wise one, so to speak… So it’s a fundamental part of it.*

[P5#FG1]

#### Establishment of a Good Therapeutic Alliance (Variant)

Participants felt that a bond with therapists could be established in this format and reported having established a good therapeutic alliance.

***Interviewer:** Did you feel that this alliance was able to develop in the same way that perhaps you would have developed it face-to-face, though, in this online version?*

***P9#FG2:** I think so.*

***P10#FG2:** I also, I mean, even though they don't know absolutely everything about me and everything that has happened to me, I think I felt that commitment, didn't I? I mean, that commitment from them to us and also from us to them and to the treatment. So, yes.*

#### Greater Difficulty in Creating the Bond (Variant)

Some other participants felt that it was more difficult to create a therapeutic bond in group therapy than in individual therapy or that this bond is different. An example of this is the following intervention:

*I also agree, it’s another type of bond, no, I don’t think it’s the bond that… […] It’s another type of bond and regarding the ease of creating it or not, I think it’s more difficult in a group because there are many people, it’s more risky eh with, with one person alone it’s much easier, isn’t it?, more risky, but well, if it works, it’s also very good.*

[P4#FG1]

### Overall Assessment of the Blended Treatment

This section includes all experiences, opinions, and assessments regarding the intervention as a whole, including its impact or effects, its advantages and disadvantages, etc.

#### Perceived Improvements and Usefulness of Learning (Typical)

Most participants highlighted their improvement after the intervention as well as the usefulness of the learning achieved during the treatment in their lives, as exemplified below:

*Ehh, in my case, I also agree with my groupmates, especially, let’s see, I had enough knowledge, I think, ehh… to practice and all that, but I hadn’t taken the time to do it, I mean, the theory was ‘ok, very good,’ but I had never taken the time, and it seemed to me that the treatment has been like an opening key to something that was already there, you know? Mm… especially the fact of continuing to practice, of saying ‘jeez, it’s fundamental…,’ like going to the gym, right? to keep practicing and so on. It has helped me a lot to become aware that… that I need it, and… and I have also changed a lot from before having started it to now, it has helped me a lot. […] I would like to add that in my case the situation hasn’t changed, I mean, there are some groupmates whose situation has changed and so on, but in my case it has not, that’s why I’m happier in life, because the situation hasn’t changed, but I’ve improved and I’m handling things much better.*

[P8#FG2]

#### Satisfaction With the Treatment (Variant)

Some participants also emphasized their satisfaction and positive feelings toward the treatment received, as can be seen in the following case:

*I feel that… you can’t cure a problem, none, in a few online sessions and that you have to work and for that what they give you are tools to achieve it individually and then, in that, I think it has helped me a lot, I think they have given me many tools to know how to manage what happens to me, more than cure me, and also… ehh well, the importance of emotions and what happens to me in general I have also learned a lot and, and well, I think that 70-80% has satisfactorily fulfilled everything what, what I expected.*

[P1#FG1]

#### Recommended Treatment (Variant)

Some participants went a step further and indicated that they would recommend the treatment to others with emotional problems.

*It is true that it requires more work and more time, but I would recommend it. The truth is that it is quite useful, and I would invite anyone who has any emotional disorder to do this treatment because the truth is that it is really useful.*

[P12#FG3]

#### Advantages (Typical)

Most participants mentioned advantages of this type of blended treatment, which combines group sessions delivered via videoconference with self-applied work through an online platform. The following excerpt is an example of an outstanding advantage:

*Well, the advantages. Mmm. I think the convenience, as my groupmates mentioned, it’s very easy to attend the sessions, very easy to access the platform, the course. […] Um, another thing. The main advantage that I enjoyed was having taken this course from another country. I wouldn’t have been able to take it. I wouldn’t have. I wouldn’t have. It doesn’t exist in Mexico. So, well, for me it’s a great advantage.*’

[P18#FG4]

#### Disadvantages (Variant)

Participants also mentioned disadvantages of this type of blended intervention, among them:

*I think if I had been alone with one of them (therapists), I think it would have been, I would have focused more on, on my problems or I don’t know… This was more, more general, a help, […] it has been very helpful, but […] it doesn’t focus on what I think my problem is.*

[P6#FG1]

### Elements That Help to Maintain Adherence

This domain includes participants’ opinions and experiences regarding the aspects of the intervention that helped them to stay motivated, continue progressing with the treatment, and promoting adherence to it.

#### Therapist Support (Variant)

Participants identified therapeutic support as a key factor that helped them maintain motivation and continue with treatment.

***Interviewer:** Did you notice any other aspects? Because it’s a procedure that involves some work at home, then the sessions, eh, there’s a lot of work there. Is there anything that’s helped you stay motivated and keep going? What do you notice the most?*

***Interviewer:** From any of the aspects of the treatment.*

***P13#FG3:** Well, obviously everything helps. Having 2 psychologists explain the topic to be addressed, of course that helps. […] I think it’s the main thing.*

#### Therapeutic Resources and Materials (Variant)

Participants identified therapeutic resources and materials, such as the modules, the videos, and the decisional balance table, as important factors for adherence.

*Well, I was going to say that I, for example, haven’t had the thought of quitting, not at all, but it’s true that there was a time when I was really swamped with exams and many other things […] So it’s true that in that moment of overwhelm, […] the table we made at the beginning of the pros and cons, I think it was, of starting treatment, of changing, and all that, helped me a lot. Well, for me, the table was, I mean, I made it to my liking, so I have it posted and that helps me a lot when I’m feeling more down or whatever, having seen it and saying, look, here’s the reminder of this. Well, that’s been very good for me.*

[P17#FG4]

#### Having Graphs That Represent Progress (Variant)

Participants highlighted that being able to see their evolution reflected in the progress graphs of the platform was a motivation element for them. The following is an illustrative example of this category:

*Seeing the progress graphically week by week is something that, at least for me, also helped motivate me to continue with the treatment*.

[P16#FG4]

#### Having Scheduled Sessions (Variant)

Participants highlighted having established sessions as an element that helped them to continue with the treatment and do the homework tasks.

*I think it has been the constancy, seeing each other, seeing each other every 3 weeks, I think it has been that, wait, today I don’t feel like doing it, okay, tomorrow neither, but in 3 weeks these people are going to ask me what… ‘what have you worked on?’ you know? So, it’s like a duty, like going to class, for me that has helped a lot. It was like I have to do my homework for college or my thesis or whatever, I have to do my psychology homework, I mean the psychologist, where I’m going, the treatment, because I started it and I have to finish it, and in 3 weeks I have to see them, so it’s been like a constant and that’s how it’s been every month.*

[P9#FG2]

#### Commitment to the Group (Variant)

Some participants also identified groupmates and commitment to the group as an element that helped them maintain motivation and not drop out of treatment, as we can see in the following case:

***Interviewer:** What elements of this treatment helped you to stay motivated, to stay connected, to keep moving forward in this treatment?*

***P6#FG1:** In my case, more than the modules and all that, the therapists and my groupmates the most.*

#### Perceived Impact of the Intervention (Variant)

Some participants highlighted that the perceived impact of the treatment, that is, noticing the effect it had on them or the progress they were making, helped them to continue with the intervention.

*In my case, what motivated me the most, even though as I mentioned before, I was behind in the course, was to see that the tools worked. Knowing that the videos and applying the tools that were mentioned in them really showed an effect on my mood, at least in the short term, I mean, because we know that this problem is long, it’s not a, you have to practice these tools in the long-term to notice a change, but a change was noticeable in the short-term, at least in the short-term. And that was quite motivating for me.*

[P18#FG4]

#### Readiness to Change (Rare)

Some participants mentioned the readiness to change and commitment to oneself as a motivating element.

*Also the motivation of wanting to change ehh yourself as a person, yourself… and knowing how to control yourself, knowing… well, to control! Okay, it’s accepting your emotions and ways of facing the things that happen to you, well, for me to have the motivation to find new, new ways of doing things, that were not the ones I used to do previously.*

[P5#FG1]

#### Having Reminder Messages (Rare)

One participant also noted how important the weekly motivational reminder messages they received were to her for continuing.

‘*Well, let’s see, in my opinion the fact that there are established sessions and also the constant, constant online reminders, I mean, by email, which I think have been very important, although subtle, but for me they have been very important.*

[P12#FG3]

### Proposals for Improvement

This domain refers to any suggestion, proposal, or idea to improve the intervention in any way from the patients’ point of view.

### Modifications to the Platform (Variant)

Participants suggested some modifications to the platform, aimed at improving the current functionality or incorporating new tools. We can see an example in the following extract:

P1#FG1: Yeah, well, my, my idea is, it’s also related to the platform, but I’ve also missed like the interaction, I mean, it was like just listening to the video, it would be interesting to do activities in umm like games… from the video and it explained to you Maria’s situation, for example, Maria has anxiety and this. Like a kind of ‘What would you do in this situation if you were Maria?’ Because that also puts you a little bit in the role of what you’re going through, although, although you don’t necessarily have anxiety, but… something like more interactive with the platform that also helps you to understand a little bit the type of decisions, or if it’s a good decision, that kind of thing, it would be interesting ehh that the platform itself motivates a little bit more and not just listening to a video.’

### Adjustments in the Configuration (Variant)

Participants proposed some adjustments regarding the pace of progress on the platform and the duration and periodicity of sessions, as exemplified below:

***Interviewer:** Do you think that the duration of the group sessions that you had, 2 hours of online sessions every 3 weeks, is adequate? That is, 2 hours every 3 weeks, did you see it as adequate or how did you feel that temporality?*

***P11#FG3:** I think it’s fine. To be there for 2 hours, ehh it’s really like attending a therapy session or even a class.*

***Interviewer:** And what about it being every 3 weeks? Did you find that spaced out enough, too spaced out?*

***P11#FG3:** Mm maybe… it depends, it depends on the subject that we were doing… more time or less time would have been better.*

### Suggested Changes Regarding the Sessions (Variant)

Some participants made suggestions about the sessions, proposing some additional sessions or that some of the sessions be held in person.

*Even though it’s online, I like it, but later, when we were doing about 2 or 3 sessions, I would also have liked it to be in-person format because well I actually miss in-person attendance, because I got used to it at the university and then when we went back to in-person, the difference was also noticeable, so, well, doing at least one or 2 in person I think it would also help a lot*.

[P1#FG1]

#### Greater Individualization of the Intervention (Variant)

Some participants emphasized the importance of personalizing or individualizing the intervention further, as we can see in this case:

***P8#FG2:** Maybe what was missing… I don’t know, a proposal also, like maybe 10 minutes, once in a while, not every week, obviously it cannot be done, to go deeper into the problem or more individual difficulties. You know? […] It’s true that there were sessions when we were asked about the problems, ‘how did you deal with them?’ and each of us talked a little bit. But, yes, I was referring maybe to one more difficulty that you have, and you can’t share with the group or whatever, no… maybe not frequently, but it would be good.*

***Interviewer:** It would be like a space for, an individual space with the therapist, right? To share the things that in the group are not…*

***P8#FG2:** Exactly.*

#### Suggestions for Improving Adherence (Variant)

Participants made proposals to improve adherence to treatment and reduce dropouts. This category includes proposals specifically linked to enhancing adherence, although some of them mention, for example, changes in the configuration or suggestions for greater individualization.

***Interviewer:*** *Great, okay. Emm can you think of any strategies that you can or we can recommend, we can include improving adherence, so that people can get to finish this treatment, prevent them from dropping out of therapy, that you want to suggest to us?*

***P3#FG1:*** *I don’t know if it could be done, or if it would be useful, but maybe ehh as a kind of weekly follow-up by the, by the therapists, even if it’s by email, asking you how the week went or… I don’t know.*

### Perspective of Dropouts

Participants who discontinued the intervention identified various reasons for dropping out. Participants mentioned treatment-related variables (lack of results, treatment-related stress, or difficulties applying the strategies), person-related variables (low involvement, lack of motivation and energy, or time constraints), and the need for other types of help (in-person or more specialized professional help). As strategies to improve adherence to the treatment, participants suggested adjustments in the format and configuration of the sessions (individual and in-person or weekly) and other aspects (less homework tasks and more time to discuss individual problems). Table 3 shows the literal responses to the dropout questionnaire.

## Discussion

### Principal Findings

The goal of this study was to explore in depth the experiences and opinions of patients who received a transdiagnostic CBT intervention for ED, delivered in a blended and group format, in order to understand their degree of acceptance and improve the future implementation of these types of interventions.

Overall, the results of this study indicate a general satisfaction with the transdiagnostic intervention, consistent with prior evidence on the feasibility and utility of blended psychological treatments [26,35]. Positive perceptions were observed across multiple dimensions, including the usability of the web platform, the perceived richness and relevance of the therapeutic contents, the added value of group format, and the perceived support provided by the therapists. Furthermore, participants generally reported perceived improvements following the intervention and emphasized the advantages of this blended format. Consequently, this intervention appears to be both feasible and well-accepted by patients for the treatment of ED. The results of this study also provide relevant information on specific measures that could be adopted in the future to further optimize treatment and minimize dropout rates.

Generally, participants reported an overall positive user experience with the platform, particularly regarding its intuitive design and the availability of multimedia materials and contents. They also appreciated the different tools offered by the platform. These results are consistent with the previous feasibility and acceptability study in which the patients included in this study participated [30]. In the aforementioned study, adequate adherence to the platform and high-excellent usability according to the System Usability Scale [36,37] scores were observed. Previous studies on other treatment protocols administered through the same web platform also reported high usability scores [38,39]. In addition, these findings align with research suggesting that ease of use, clarity, usability, attractive appearance, and perceived utility are critical factors in the acceptance and engagement with digital mental health interventions [40,41]. Participants also identified some technical difficulties with the platform, such as progress within each module not being saved and disconnection of the platform after a period of inactivity that required them to log in again. However, this feature was intentionally implemented as a security measure to protect users’ privacy if the platform was accidentally left open. A future challenge will be to maintain robust security standards that enable patients to use digital devices confidently and safely [42] without compromising usability and acceptability. Technical problems have also been reported in other studies of blended interventions [12,26,27], suggesting that the difficulties identified in this study are common in this field.

The mixed evaluations of pace of progress on the platform reflect a common challenge in online and group interventions: balancing standardization with individualization. While participants were generally satisfied and considered the weekly pace adequate, others wished for a more flexible rhythm, particularly for modules involving complex skills such as emotional avoidance or acceptance. Prior literature suggests that personalizing the configuration of blended treatments may enhance adherence and treatment benefits [43]. As the systematic review by Erbe [14] indicates, there is no single optimal ratio between face-to-face and online components in blended interventions; thus, patient-specific customization may be key. The length of the sessions was considered appropriate by most participants in focus groups, although a few felt they were slightly long. There were also differing opinions on session frequency; some found the timing adequate to work on the content of the modules, while others would have preferred more frequent sessions or less time between them. These findings are in line with the heterogeneity of session lengths reported in the literature. For example, Osma et al [44] implemented 2-hour unified protocol group sessions, while Schuster et al [45] noted that therapists were concerned that 90-minute weekly sessions did not allow enough time for discussion. This variability of configurations in the literature highlights the need to customize the specific combination of in-person and online elements, as well as the timing. This personalization of the intervention has been emphasized by Fernández-Álvarez et al [12] and Patel et al [13], especially within transdiagnostic frameworks where symptom profiles and levels of functional impairment can vary significantly. Although not asked directly, a couple of focus group participants talked about the duration of the treatment. While one participant felt that the duration had allowed them to track progress month by month, another participant believed that it could cause a loss of motivation. Interestingly, participants mentioned personal variables that had an impact on the optimal fit of the planned configuration. These insights suggest that individual factors, such as workload, time management challenges, or fluctuating symptoms, may impact the optimal engagement and highlight the value of adapting to the patient’s life circumstances.

Participants highlighted the appropriateness and richness of the content. They valued the material positively, indicating that it is a very comprehensive program and suggesting that everyone should have access to the modules. They stated that the content was well explained and relevant to their issues. They also pointed out which content had been most useful for their specific cases. Mindfulness was the strategy mentioned by more participants, followed by activities belonging to the positive affect component—such as value-based behavioral activation or the identification of psychological strengths—and emotional avoidance and emotion-driven behavior modules. It may be thought that patients could be reluctant to adopt strategies like mindfulness, as avoidance is a common trait among people with ED [46]. Therefore, it is very important to note that participants responded positively to mindfulness and felt it was very helpful, as it was the strategy most frequently mentioned. They also highlighted the exposure component, cognitive flexibility, and relapse prevention as other useful strategies, providing a comprehensive overview of the entire program. Overall, these results align with previous quantitative findings. When participants were asked how useful each module was on a scale from 0 to 10, the average scores were 7 or higher for all modules [30]. Another important point from participants’ feedback was the variability in prior knowledge and its impact during treatment. Some participants felt unsure about which strategies to use in specific situations, while others sometimes found the content repetitive due to their previous familiarity with similar material. Previous research emphasizes the need to tailor the complexity of therapeutic content to participants’ prior knowledge and learning styles [47,48]. These findings suggest the value of preassessment tools to gauge prior familiarity with CBT concepts, which could help guide each patient’s pacing or personalized use of modular content.

Regarding the group format, participants noted some limitations and expressed initial reluctance toward this approach. However, most participants recognized positive aspects, such as learning from others and feeling a bond with the group. This aligns with literature highlighting the therapeutic value of shared experiences, group cohesiveness, and peer learning among individuals with similar issues [20,49,50]. Numerous studies on the acceptability of transdiagnostic interventions report good acceptance and high treatment satisfaction, and this is held in a group setting [51,52]. In terms of videoconferencing, participants identified limitations like potential connection issues and challenges in finding a private space. However, they also highlighted several advantages, such as the convenience of joining sessions from anywhere and the fact that emotions surfaced in the same way through videoconferencing as in face-to-face therapy. These benefits and additional advantages of this form of psychotherapy have been noted in previous literature [49,53]. This research indicates that, in general, patients have a positive attitude toward videoconferencing as a therapy option [54-56]. In line with this, some participants in this study reported satisfaction with the sessions and positively valued the dynamics of group interactions. However, despite these benefits, some participants expressed a personal preference for individual or in-person therapy. This preference was one of the recruitment challenges faced in the treatment feasibility study [30]. Previous literature has also reported that some individuals prefer in-person groups [49], and many patients with ED often favor individual face-to-face therapy [57]. Therefore, it is crucial to explore ways to make more acceptable those interventions that enhance the cost-benefit balance.

Most participants highlighted the qualities of the therapists and their availability, such as their explanations, their empathy and closeness, their expressiveness, or their response to doubts that arose, among other issues. Additionally, in general, participants emphasized the importance of the therapist’s role, indicating that it was fundamental and that without therapists they could not have done it. Likewise, they reported having established a good alliance with the therapists in the same way as in classical psychotherapy. This is consistent with previous studies in which the importance of support from therapists was highlighted [12,13]. The establishment of a strong therapeutic alliance, even in an online group format, was reported by several participants, consistent with previous research demonstrating the feasibility of alliance building in digital contexts [58]. Furthermore, research shows that patients and therapists experience a good therapeutic alliance with videoconferencing therapy, comparable to that obtained with face-to-face therapy [55,59-61]. Studies on blended interventions also show that it is possible to establish a good therapeutic alliance between therapists and patients in this format [26,35].

Participants identified several general advantages of the blended intervention they received, notably temporal flexibility and, particularly, spatial flexibility, which allows treatment to be accessed from anywhere without the need to travel. Additionally, the combination of online modules and sessions was highlighted as a positive aspect, with both elements considered useful. These advantages have been noted in other studies of blended interventions. For instance, in the study by Etzelmueller et al [26], patients also emphasized the spatial flexibility and convenience of working from home, and they found the combination of online modules and synchronous video-based sessions to be beneficial. The main disadvantage identified by participants in focus groups was the more general nature of the treatment, which limits in-depth exploration of individual circumstances or specifics. This is related to the transdiagnostic nature of the intervention and likely its group format. Despite this, most participants reported perceived improvements and the usefulness of the skills learned in their daily lives, noting that they have discovered methods that work for them, that they can better cope with difficult situations or their own symptoms, or that they feel better overall. This is consistent with the preliminary efficacy found in our previous study [30], where significant reductions in anxious and depressive symptoms were obtained at posttreatment and follow-up compared to baseline, and where significant changes were found in other clinical measures, such as negative affect, neuroticism, emotion regulation, or quality of life. In other blended interventions mentioned above [26], patients also reported positive treatment effects. Focus group participants also expressed satisfaction with the intervention, and some even said they would recommend the treatment to others with ED. This is consistent with the quantitative satisfaction reported in our previous study, where patients showed high satisfaction with the treatment [30]. It is also in line with previous research, which generally shows positive opinions [26,27] and high satisfaction with blended interventions [16].

Regarding the domain related to elements that helped them maintain adherence, the most frequent category was therapeutic support, which once again highlights the importance of the therapist’s presence. The existence of scheduled sessions and the commitment to groupmates were mentioned, which also appears to reinforce the importance of support and guidance from therapists as well as one of the advantages of the group format [20,21]. Other important points were the therapeutic materials themselves and the effects of the treatment on the patients, both in terms of their personal experience of improvement and the possibility to see their progress reflected in the platform’s graphs. In addition, although less frequently mentioned, some participants described internal factors, such as readiness to change and self-commitment, pointing to the role of intrinsic motivation. Taken together, these findings suggest that successful adherence in blended formats likely results from a combination of external (therapist, structure, characteristics, and content of treatment) and internal resources (motivation and self-management), supporting recent models of digital intervention engagement [62]. Future adaptations might benefit from integrating dynamic feedback systems and tailored support strategies to further optimize adherence.

With regard to this, during focus groups, participants suggested incorporating some changes to the platform, such as a discussion forum or a greater degree of interactivity. They also proposed adapting the configuration to the specific modules to be worked on or making the sessions more frequent, or even some of them in person. In addition, they proposed a greater individualization of the intervention, suggesting creating brief individual spaces with the therapist or combining some individual sessions with the current intervention. They also made proposals to improve adherence to treatment, some of which were also related to more individualized follow-up or leaving less space between sessions. Thus, the importance for patients of the presence of the therapists throughout the intervention and the customization of treatment is once again evident.

Finally, participants who dropped out of treatment provided reasons for dropping out and suggested some strategies to improve treatment adherence. Although they did not complete the program, we considered it important to include their experiences to enrich understanding of the intervention’s acceptability and identify potential barriers to engagement. Similar to what was found in previous studies [12], participants mentioned that the treatment was not working for them, that they had difficulty applying the strategies learned, or that they identified the need for other types of assistance, such as in-person or more specialized help. The relevance of addressing these aspects has been previously evidenced because noncompletion of tasks generates dissonance, may lead to increased discouragement, and is related to treatment success [27]. Dropout participants proposed changes regarding the format and configuration of the sessions with the aim of improving adherence to treatment, suggesting that they could be individual, in-person, or held weekly. They also believed that having more time to discuss individual problems in sessions or having fewer homework assignments could help to increase adherence. It has been shown that patients who engage more with homework assignments in online CBT interventions achieve a greater reduction in anxiety and depression symptoms [63], so it would be important to assess patients’ engagement in this regard to be able to implement preventive strategies. Several of the identified barriers and proposed suggestions for improvement coincide with aspects mentioned by participants in the focus groups, which reinforces the value of the findings obtained. Taken together, the importance of focusing on the personalization of treatment emerges.

Although this format may not be suitable for all individuals, the findings from the feasibility study [30], together with the qualitative results from this study, support the viability and acceptability of this blended intervention, which also appears to have clinical utility. Additionally, it may help to overcome some of the disadvantages of fully face-to-face and fully self-administered formats and may be a good resource to facilitate access to EBT for a broader population. This is particularly relevant in the context of public health systems such as the Spanish NHS, where there are long waiting lists and sessions occur infrequently. Therefore, a key recommendation for future research and clinical practice would be to explore which patient profiles and under what circumstances this type of blended intervention is most effective [43], as well as to identify cases where alternative formats may be more beneficial.

### Limitations

This study has several limitations. One of them is that retrospective responses pose a methodological limitation due to possible recall bias. However, this is a common limitation in qualitative research and not specific to this study. Nonetheless, the focus groups were conducted shortly after the end of the intervention, which likely reduced the extent of this bias. An additional limitation is that 2 of the participants in the focus groups hardly spoke during the interview, resulting in very few ideas from them, which made it difficult to reach the “General” category. Another limitation is that very few participants who dropped out of treatment completed the dropout questionnaire, so formal qualitative analyses could not be conducted. Finally, it would be advisable for future studies to be conducted with people treated in other settings, such as the Spanish NHS, in order to increase the generalizability of the results.

Despite the aforementioned limitations, this study contributes to completing the established conclusions regarding the feasibility, acceptability, and preliminary efficacy of the intervention studied [30]. Furthermore, to our knowledge, it is the first qualitative study to explore the opinions of patients (completers and noncompleters) who have received a transdiagnostic intervention applied in group and blended formats, thus contributing to the development of this novel format.

### Conclusions

This study reinforces the feasibility and acceptability of a blended transdiagnostic group treatment for ED. These qualitative findings provide valuable insight into patient experiences, perceived benefits, and areas for improvement while also offering guidance for future research and implementation. This study supports this innovative format as a potentially scalable, acceptable, and effective option within mental health services.

## Supplementary material

10.2196/86016 Multimedia Appendix 1 Interview script.

10.2196/86016 Checklist 1 COREQ checklist.

## Abbreviations

CBT: cognitive behavioral therapy
COREQ: Consolidated criteria for reporting qualitative research
CQR: consensual qualitative research
EBT: evidence-based treatment
ED: emotional disorder
NHS: National Health System
