# Supplementary material for: Exploring Patients’ Experiences in a Blended Transdiagnostic Group Treatment: Qualitative Study
Source: JMIR Hum Factors. 2026 Jul 6;13:e86016. doi: 10.2196/86016 (PMC13335943; doi:10.2196/86016)
Supplement: Multimedia Appendix 1 [file humanfactors-v13-e86016-s001.docx]

**SUPPLEMENTARY MATERIALS**

**Multimedia Appendix 1 – Interview script**

1. How has your experience with the online treatment platform (access, use, modules, review and progress tools, etc.) been?
2. Do you think progressing at a pace of one module per week is adequate?
3. What do you think about the content of the treatment modules? Are there some modules that have helped you more than others? Which ones and why?
4. Do you think the length of the group sessions is appropriate? And the frequency (every three weeks)?
5. How do you feel about the fact that the sessions are conducted in groups? And about the fact that they are conducted via videoconference?
6. How do you value having support from the therapists in the online group sessions?
7. Do you think the therapeutic alliance can be developed in the same way in this type of therapy as in traditional face-to-face therapy?*
8. What do you think are the advantages of this blended treatment (self-administered + online group sessions) compared to traditional face-to-face psychotherapy? And what about the disadvantages?
9. To what extent do you think the treatment has helped you to solve the problem for which you sought help?
10. What elements of the treatment helped you stay motivated and continue with the treatment?
11. Can you think of any strategies to improve treatment adherence and reduce dropout rates in this type of therapy? Which are they?
12. What is your overall satisfaction with the treatment as a whole? Would you recommend this treatment to other people with emotional disorders?

**NOTE*. In question 7, the term ‘therapeutic alliance’ was explained to the participants before the question was asked.
